# Application and practice of a step-by-step method combined with case-based learning in Chinese otoendoscopy education

**DOI:** 10.1186/s12909-021-02513-1

**Published:** 2021-02-04

**Authors:** Fanqin Wei, Qiyang Sun, Zili Qin, Huiwen Zhuang, Guangli Jiang, Xuan Wu

**Affiliations:** 1 Department of Otorhinolaryngology, Head and Neck Surgery, The First Affiliated Hospital, Sun Yat-sen University, 2nd Zhongshan Road 58#, Guangdong 510080 Guangzhou, People’s Republic of China; 2 Institute of Otorhinolaryngology Head and Neck Surgery, Sun Yat-sen University, Guangdong 510080 Guangzhou, People’s Republic of China; 3 Guangzhou Key Laboratory of Otorhinolaryngology, Guangdong 510080 Guangzhou, People’s Republic of China

**Keywords:** Otoendoscopy, Case‐based learning, Step‐by‐step method, Operation teaching

## Abstract

**Background:**

Standardized training allows more physicians to master otoendoscopic surgery. However, the lecture-based learning (LBL) applied in otoendoscopy teaching may not be conducive to training students in clinical thinking and surgical ability. It is necessary to explore innovative methods for otoendoscopy teaching. This study aimed to determine the effect of a step-by-step (SBS) method combined with case-based learning (CBL) in otoendoscopy teaching.

**Methods:**

Fifty-nine physicians who participated in otoendoscopy training were selected as the study subjects and randomly divided into two groups (A and B). Group A underwent training with the SBS & CBL method, while Group B underwent training with the LBL & CBL method. The effects of these two methods for otoendoscopy training were compared by evaluation of professional skills and questionnaires before and after the training.

**Results:**

Proficiency in otoendoscopic anatomy and grades for both professional knowledge and otoendoscopic skills were significantly higher in Group A than in Group B(*P* < 0.05). In terms of learning interest, surgical ability, acting capacity during surgery, reducing surgical complications, and satisfaction with learning experience, all responses from Group A were better than those from Group B(*P* < 0.05).

**Conclusions:**

The SBS & CBL method may help to improve ability in otoendoscopic surgery and clinical thinking and appears suitable for endoscopy teaching.

## Background

Developments in modern minimally invasive ear surgery are closely related to the development and application of otoendoscopy. In recent years, with the upgraded otoendoscopes and surgical instruments available, the advantages of otoendoscopic minimally invasive surgery have become increasingly prominent [1–3]. Compared with traditional ear surgery that introduces skin incisions beside the ears, otoendoscopic minimally invasive surgery avoids open incisions and thus significantly shortens both the operation time and the wound healing time. Consequently, it is of great practical significance to carry out standardized training, thereby allowing more otologists to master otoendoscopic surgery and reducing and avoiding surgical complications.

Published literature has mainly focused on the advantages and disadvantages of otoendoscopic surgery and its surgical indications [4, 5]. However, techniques for how to carry out standardized otoendoscopic surgery have rarely been reported. The current and most widely used teaching method is traditional lecture-based learning (LBL), which is a teacher-led method. Large amounts of professional knowledge and clinical experience are introduced to students who are passive receivers of such teaching. During the teaching process, the otoendoscopic surgery is completed by senior physicians and students have few chances to perform operations, which may reduce their interest and enthusiasm to learn. As a result, their surgical ability may not show marked improvement. Therefore, LBL might be less conducive to training students in clinical thinking and practical ability, and the teaching strategy requires further reformation and exploration.

In the present study, we adopted a step-by-step (SBS) method combined with case-cased learning (CBL) for endoscopy teaching. The SBS method involves step-by-step completion of the teaching process, while CBL is a case-based, problem-based, student-oriented, and teacher-led teaching model. By comparison with the traditional LBL, the effects of the two different teaching methods in otoendoscopy training were analysed for their ability to improve the quality of clinical teaching.

## Methods

### Teaching implementation

#### SBS & CBL method

There were three steps in the SBS & CBL method: otoendoscopic operation, temporal bone anatomy training, and otoendoscopic surgery practice. First, under the guidance of teachers, training in operative procedures, including otoendoscopic examinations, treatments, and post-operative examinations, was conducted in an outpatient room for 4 weeks. Second, training in otoendoscopic anatomy was conducted for 4 days using temporal bone specimens. Under the guidance of teachers, students learned to identify various anatomical signs in the middle inner ear by otoendoscopy, including vestibular window, round window, facial nerve crypt, auditory ossicle, cochlea, and inner auditory canal. They also performed practice to master the common methods and protocols in otoendoscopic surgery. Third, clinical surgery was carried out in the otology department. Under the guidance of teachers, students took care of patients during the perioperative period combined with the CBL method. Before surgery, students were required to report typical cases combined with preoperative examinations such as tympanograms, hearing tests, and mastoid CT. By considering the latest research in similar cases, teachers and students discussed the surgical methods, risks, preoperative conditions, and key points in preoperative conversations. During surgery, the teachers explained the operation skills and precautions, and students could ask questions about potential intraoperative situations. After surgery, students completed the operation records, which were revised by the teachers in due course. In post-surgery rounds, students analysed the causes of common complications and proposed treatment suggestions from the perspective of anatomy and pathophysiology, and teachers corrected the analysis deviations to provide standardized post-operative treatment processes.

#### LBL & CBL method

Studies were arranged for in-patients in the otology department. All techniques for otoendoscopic surgery and temporal bone anatomy were imparted to students on the basis of lectures and teaching ward rounds, as the major difference between the LBL and SBS methods. Subsequently, the CBL method was carried out as described for the third step of the SBS & CBL method.

### Assessment methods

#### Self‐assessment of proficiency in otoendoscopic anatomy

Based on the method reported by Edmond et al. [6], we prepared an assessment form for the effect of training in otoendoscopic surgery. Students filled in the form anonymously before and after their training in temporal bone anatomy and training in otoendoscopy. Proficiency in otoendoscopic anatomy and sign identification in the middle inner ear were assessed by scores ranging from 0 to 10 points using a Visual Analogue Scoring (VAS) method (completely unskilled: 0 points; unskilled: 1–3 points; skilled: 4–6 points; proficient: 7–9 points; expert: 10 points). The Cronbach’s alpha was 0.825.

#### Examination grades

Before and after their training, the students in both groups took examinations on professional knowledge and surgical skills (100 points for each). The professional knowledge examination covered common ontological diseases, otoendoscopic anatomy and surgical complications, analysis and treatment of typical cases, key points of otoendoscopic surgery, and post-operative treatment procedures. Assessment of surgical skills included otoendoscopic treatments and otoendoscopic operations (such as external ear canal flap separation, restoration, and haemostasis in surgery). Surgical skills were graded based on the otoendoscopic operation procedures, standardization, proficiency, and surgical effect.

#### Questionnaires

After the otoendoscopy training, anonymous surveys were conducted in both groups to assess the training effects of the two methods. The questionnaires included the following evaluation items for both training methods: learning interest, surgical ability, acting capacity during surgery, reduction in surgical complications, clinical thinking, learning pressure, and satisfaction with training method. The evaluations were graded as follows: 1 = dissatisfied; 2 = neutral; 3 = satisfied.

### Statistical analysis

SPSS 22.0 software (SPSS Inc., Chicago, IL, USA) was used to perform data analyses. Data with a normal distribution were presented as mean ± standard deviation and a *t*-test was used for comparisons between the two groups. Count data were presented as case count and percentage and differences between the two groups were evaluated by the chi-square test. Categorical data were compared using the Kruskal–Wallis test. All tests were two-sided, and values of *P* < 0.05 were considered to indicate statistical significance.

## Results

### Comparison of participants between the two groups before training

Fifty-nine physicians who participated in otoendoscopy training at the Department of Otorhinolaryngology in the First Affiliated Hospital of Sun Yat-sen University between January 2018 to December 2018 were selected as the study subjects. A single-blinded randomisation was conducted, with 28 participants allocated to Group A (17 men, 11 women) and 31 participants allocated to Group B (18 men, 13 women). Each group was trained for 6 months. The SBS & CBL method was adopted in Group A, and the LBL & CBL method was adopted in Group B. The teachers in both groups were senior otologists and deputy chief physicians. All students had some basic knowledge of otoendoscopic operations, but had never performed otoendoscopic surgery independently or participated in similar training. The students were informed about the method, purpose, and significance of the study, and volunteered to participate. There were no significant differences between the two groups in terms of sex, age, educational background, hospital level, and experience in otoendoscopic operations (*P* > 0.05; Table 1).

**Table 1:** Characteristics of the students in Group A and Group B

Characteristics	GroupA(*n* = 28)	GroupB(*n* = 31)	*P* value
Gender			0.84
Male	17(60.7 %)	18(58.1 %)	
Female	11(39.3 %)	13(41.9 %)	
Age (Yrs.)	28.7 ± 1.6	29.3 ± 1.5	0.14
Degree			0.78
Bachelor	11(39.3 %)	12(38.8 %)	
Master	15(53.6 %)	18(58.1 %)	
PhD	2(7.1 %)	1(3.1 %)	
Hospital level			0.90
Class III hospital	10(35.7 %)	14(45.2 %)	
Class II hospital	18(64.3 %)	17(54.8 %)	
Experience in otoendoscopic operation after graduation (Physician)			0.70
0~1 year	8(28.6 %)	6(19.4 %)	
1~2 years	17(60.7 %)	21(67.7 %)	
> 2 years	3(10.7 %)	4(12.9 %)	

### Evaluation of anatomical identification and surgical skills before and after training

To assess the effects of the otoendoscopic training methods, we evaluated the proficiency in otoendoscopic anatomy and surgical skills for otoendoscopic operations. After the otoendoscopy training, we found that the proficiency in otoendoscopic anatomy and surgical skills for otoendoscopic operations were significantly improved in both groups. Importantly, the examination grades in Group A (SBS & CBL) were significantly higher than those in Group B (LBL & CBL) in all aspects (*P* < 0.05), even though there were no significant differences between the two groups before the training (*P* > 0.05; Table 2). These results indicated that the SBS & CBL method was more conducive to improving the proficiency in otoendoscopic anatomy and surgical skills for otoendoscopic operations during teaching.

**Table 2:** Evaluation of surgical skills and VAS grades for anatomical identification of the middle inner ear before and after the otoendoscopy training (points; mean ± standard deviation)

Items	Group A(*n* = 28)	Group B(*n* = 31)	*P* value
Overall proficiency in using otoendoscope			
Before	4.1 ± 0.4	4.3 ± 0.5	0.09
After	9.2 ± 0.5	6.9 ± 0.6	< 0.01
Proficiency in using otoendoscope			
Before	3.7 ± 0.4	3.3 ± 0.5	0.78
After	8.8 ± 0.5	6.8 ± 0.5	< 0.01
Proficiency in otoendoscopic surgery			
Before	4.8 ± 0.4	4.7 ± 0.5	0.40
After	8.4 ± 0.6	6.6 ± 0.5	< 0.001
Proficiency with middle ear structure			
Before	3.6 ± 0.4	3.7 ± 0.5	0.40
After	8.3 ± 0.6	6.8 ± 0.4	0.01
Proficiency with auditory ossicles structure			
Before	5.1 ± 0.5	4.9 ± 0.6	0.17
After	8.5 ± 0.4	7.3 ± 0.4	< 0.001
Proficiency with vestibular and round windows structure			
Before	4.5 ± 0.6	4.3 ± 0.4	0.13
After	8.6 ± 0.5	7.5 ± 0.5	< 0.01
Proficiency with horizontal segments of tympanic nerve and facial nerve			
Before	4.1 ± 0.6	4.3 ± 0.4	0.13
After	9.3 ± 0.7	7.8 ± 0.5	< 0.001
Proficiency with inferior tympanic and eustachian tube tympanic mouth			
Before	4.3 ± 0.5	4.6 ± 0.6	0.43
After	8.9 ± 0.5	7.5 ± 0.5	< 0.001
Proficiency with tympanic sinus and facial nerve crypt			
Before	2.2 ± 0.5	2.4 ± 0.5	0.13
After	6.7 ± 0.4	5.4 ± 0.4	< 0.001

### Anatomical identification of the temporal bone in Group A

**Fig. 1:**
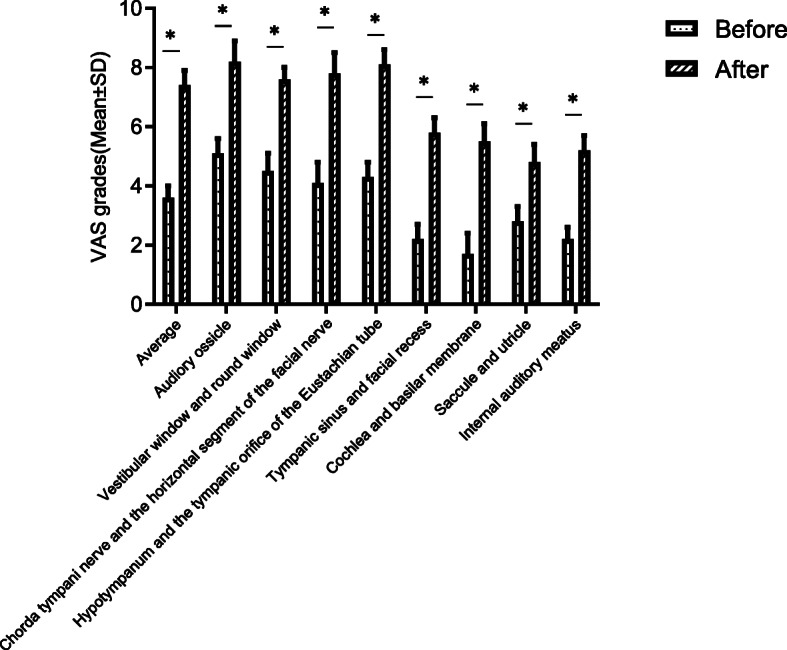
VAS grades in anatomical proficiency before and after the temporal bone anatomy training in Group A(*n*=28). The training significantly improved the proficiency in otoendoscopic anatomy in 9 aspects. *p<0.01, by a *t*-test

We suspected that the difference in training effect between the two groups may be due to the otoendoscopic anatomy training for the temporal bone in Group A. Therefore, we further graded the anatomical identification of the temporal bone before and after the training in Group A. The VAS grades (mean ± standard deviation) were: average (before: 3.6 ± 0.4; after: 7.4 ± 0.5); auditory ossicle (before: 5.1 ± 0.5; after: 8.2 ± 0.7); vestibular window and round window (before: 4.5 ± 0.6; after: 7.6 ± 0.4); chorda tympani nerve and horizontal segment of the facial nerve (before: 4.1 ± 0.7; after: 7.8 ± 0.7); hypotympanum and tympanic orifice of the eustachian tube (before: 4.3 ± 0.5; after: 8.1 ± 0.5); tympanic sinus and facial recess (before: 2.2 ± 0.5; after: 5.8 ± 0.5); cochlea and basilar membrane (before: 1.7 ± 0.7; after: 5.5 ± 0.6); saccule and utricle (before: 2.8 ± 0.5; after: 4.8 ± 0.6); and internal auditory meatus (before: 2.2 ± 0.4; after: 5.2 ± 0.5), the training significantly improved the proficiency in otoendoscopic anatomy in 9 aspects (*P* < 0.05; Fig. 1). These results indicated the importance of otoendoscopic anatomy training for the temporal bone.

### Skill assessment before and after training

**Fig. 2:**
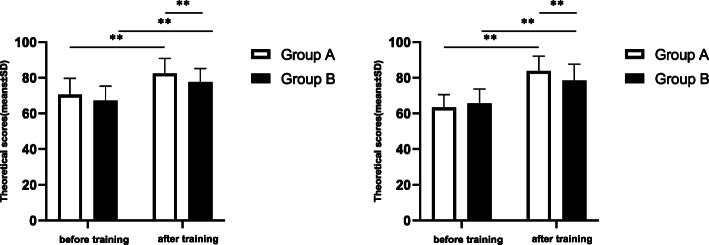
Evaluation of training effects. The theoretical and technical examination scores for the two groups of students before and after ear endoscopy training are shown. Through skill assessments, we compared the grades of the students for both professional knowledge and surgical skills (100 points for each). *P<0.05, **P<0.01, by a *t*-test

Through skill assessments of the students, we compared the training effects objectively. The results revealed no significant differences in professional knowledge and surgical skills for otoendoscopic operations between the two groups before the training (P > 0.05). After the training, the grades for the students in both groups were significantly higher (*P* < 0.05). Importantly, the grades for both professional knowledge and surgical skills for otoendoscopic operations in Group A were significantly higher than those in Group B (*P* < 0.05; Fig. 2), indicating that the SBS & CBL method was more conducive to improving professional knowledge and surgical skills for otoendoscopic operations in teaching than the LBL & CBL method.

### 3.5 Questionnaire results

A total of 59 questionnaires were sent out and recovered, giving a recovery rate of 100 %. The satisfaction rate in Group A was high at 82.1 %, and the self-evaluated satisfaction rates for clinical thinking improvement were 85.7 % in Group A and 83.9 % in Group B. Group A showed significantly better responses than Group B for improved learning interest, surgical ability, intraoperative acting capacity, reduction in surgical complications, and satisfaction with teaching method (*P* < 0.05, by the Kruskal–Wallis test; Table 3).

**Table 3:** Feedback on the two teaching methods from students

Questions	Group A(*n* = 28)	Group B(*n* = 31)	*P* Value
1. This method increases learning interest			0.03
1	2(7.1 %)	8(25.8 %)	
2	7(25.0 %)	10(32.3 %)	
3	19(67.9 %)	13(41.9 %)	
2. This method improves the ability of surgical operation			< 0.001
1	0(0 %)	7(22.6 %)	
2	6(21.4 %)	13(41.9 %)	
3	22(78.6 %)	11(35.5 %)	
3. This method improves the acting ability during surgery			0.03
1	2(7.1 %)	7(22.6 %)	
2	7(25.0 %)	11(35.5 %)	
3	19(67.9 %)	13(41.9 %)	
4. This method reduces surgical complications			0.008
1	3(10.7 %)	8(25.8 %)	
2	5(17.9 %)	12(38.7 %)	
3	20(71.4 %)	11(35.4 %)	
5. This method improves clinical thinking ability			0.81
1	0(0 %)	1(3.2 %)	
2	4(14.3 %)	4(12.9 %)	
3	24(85.7 %)	26(83.9 %)	
6. This method reduces the learning pressure			0.67
1	8(28.6 %)	7(22.6 %)	
2	10(35.7 %)	12(38.7 %)	
3	10(35.7 %)	12(38.7 %)	
7. Are you satisfied with the teaching mode			0.002
1	0(0 %)	7(22.6 %)	
2	5(17.9 %)	10(32.3 %)	
3	23(82.1 %)	14(45.1 %)	

Notes: 1 = dissatisfied; 2 = neutral; 3 = satisfied

## Discussion

Traditionally, the teacher-led LBL method was commonly used in otoendoscopy training to deliver clinical experience and introduce surgical operations. However, this method may restrict the subjective initiative of students, thus limiting their clinical thinking and improvement. Therefore, to improve the effect of otoendoscopy teaching, it is necessary to reform the previous teaching methods.

The CBL method originated from Harvard Medical School and Harvard Law School in the United States at the beginning of the twenty-first century. During the teaching process, teachers select typical cases and then guide students to conduct in-depth analyses and discussions, with the purpose of encouraging their curiosity and stimulating their interest in learning [7]. Students learn relevant knowledge from typical cases and improve their clinical thinking through case interpretation. In the CBL method, teachers do not simply introduce clinical knowledge while students passively receive information. Instead, the method closely integrates clinical practice into the teaching process. As a heuristic teaching method, CBL has received extensive attention in clinical teaching in recent years [8–12]. In the present study, the CBL method was adopted in both groups. It was teacher-led and student-oriented: the pre-operative and post-operative conditions of typical otoendoscopy cases were discussed to fully encourage the subjective initiative of students so that they could effectively master the standardized perioperative method in otoendoscopic surgery. The results showed that the clinical thinking of students in both groups was greatly improved by this approach.

However, the CBL method also has some limitations for improving the surgical ability. Qualified otoendoscopic physicians require repeated and intensive surgical training. However, because the CBL method lacks experience in otoendoscopic surgery, students cannot match the CT and MR images to what they see during operations. They are also unfamiliar with the anatomical signs and surgical details, resulting in no affirmation from teachers. The teachers actually perform the surgery and explain their actions independently. Students may have hardly any chances to operate and improve their practical ability. Therefore, in otoendoscopy teaching, we need to combine the CBL method with other methods and increase surgical training based on discussions and explanations to effectively improve the teaching effect.

Compared with training in other specialties, such as urology and gastroenterology, otoendoscopy training has its own special characteristics. Because the structure and anatomy of the middle inner ear are complicated with limited space for surgery, the learning cycle in otoendoscopy is longer, and can only be taught in a step-by-step manner. The SBS method is a teaching concept for endoscopic surgery that has emerged in recent years [13]. It has been applied to multiple clinical disciplines, and achieved good teaching effects [14–19].

In the present study, the SBS method was used in Group (A) Students first trained in clinical otoendoscopic examinations and treatments, which allowed them to become familiar with one-handed operations, otoendoscopes and commonly used instruments, anatomical signs, and operation tips. Second, temporal bone specimens were used for anatomical training in the middle inner ear. This improved not only their surgical skills, but also their identification ability. It also helped to familiarize the students with common surgical procedures in otoendoscopy. Finally, clinical practice was performed in the training. At this time, the students were very familiar with the shape, size, and adjacency of the middle inner ear structures under otoendoscopy. With explanations from the teachers during the operation, students could pay attention to many surgical details and ask corresponding questions in discussions with the teachers. Through these experiences and courses, students performed otoendoscopic surgery in a step-by-step manner, which may help to reduce surgical complications, remove psychological barriers in surgery, and enhance self-confidence in learning. After a considerable amount of otoendoscopy training, the performance of the students was confirmed by their teachers. The students could complete part or entire operations under the guidance of their teachers. After the SBS method, the surgical ability in Group A was significantly improved with significantly better grades in skill assessments compared with that in Group (B) The questionnaire survey showed that the surgical ability and acting capacity in Group A were greatly improved. The satisfaction rate in Group A was 82.1 %, which was better than that in Group B. It is considered that the SBS method can effectively improve students’ otoendoscopy performance and is conducive to stimulating their enthusiasm for learning.

In summary, the SBS & CBL method in endoscopy teaching improved students’ interest in learning, better trained their abilities in otoendoscopic surgery and clinical thinking, and effectively improved the quality of teaching. Otoendoscopy is a minimally invasive surgical technique that has emerged and flourished in the Chinese otology community in recent years. However, no related teaching methods have been published. Our study explored the teaching methods for otoendoscopic surgery and obtained the preliminary research conclusion that the SBS & CBL method is suitable for endoscopy teaching. In the present study, a single-blind randomisation was conducted to reduce the occurrence of bias. The learning process and time for the two groups of students did not overlap. When the teachers introduced the teaching methods to the students, the teachers’ own value orientation for the teaching methods should not be brought into the teaching. Nevertheless, the VAS and questionnaire survey evaluation methods used in this study were subjective standards and the sample size was small; therefore, there may be bias issues such as whether the students were serious about and valued the evaluation and scoring items, which are shortcomings of the study. In addition, the observation time for the teaching practice effects in this research was short. It is necessary to further expand the sample size and conduct multi-centre clinical teaching practice studies in the future to confirm the practicability and advantages of this teaching method, with a view to further promoting its application.

## Conclusions

The SBS & CBL method is conducive to improving the ability in otoendoscopic surgery and clinical thinking and appears suitable for endoscopy teaching.

## Data Availability

All data are available upon request from the authors.

## Abbreviations

SBS: Step-by-step method
CBL: Case-based learning
LBL: Lecture-based learning
CT: Computed tomography
MRI: Magnetic resonance imaging
VAS: Visual analogue scale
